# Educational strategies to enhance dental students’ social responsibility: a scoping review

**DOI:** 10.1186/s12909-025-07941-x

**Published:** 2025-10-02

**Authors:** Eun-ju Park, Yoon Min Gil

**Affiliations:** 1 Dental Research Institute, Seoul National University, 101 Daehak-ro, Jongno-gu, Seoul, South Korea; 2 Department of Dental Education, School of Dentistry, Seoul National University, 101 Daehak-ro, Jongno-gu, Seoul, South Korea

**Keywords:** Community-based education, Dental education, Dental student, Social responsibility

## Abstract

**Background:**

Fostering socially responsible dentists is a fundamental goal of dental education. This scoping review aimed to identify and analyze educational programs designed to enhance dental students’ social responsibility.

**Methods:**

In January 2025, a comprehensive literature search was conducted across PubMed, Scopus, and Embase. Studies were included if they involved undergraduate dental students and described educational interventions explicitly aimed at promoting social responsibility. Key characteristics, assessment methods, and reported outcomes were extracted and synthesized. Based on the findings, a conceptual framework was developed using Engeström’s activity theory.

**Results:**

Sixteen studies met the inclusion criteria. Most programs adopted a community-based educational approach, involving dental or non-dental activities. Reflective writing was incorporated as a pedagogical tool. Program durations ranged from single-day interventions to multi-year curricular integrations. Surveys and reflective writing were the primary assessment methods and consistently indicated improvements in students’ awareness of social issues, sense of responsibility, and intentions to serve underserved populations. Based on these findings, a conceptual framework was proposed to illustrate how community-based programs can support the development of social responsibility in dental students.

**Conclusion:**

This review identified community-based education as essential for developing dental students’ social responsibility, emphasizing the need for structured community engagement programs combined with critical reflection in dental curricula.

**Supplementary Information:**

The online version contains supplementary material available at 10.1186/s12909-025-07941-x.

## Background

Dentists are granted the right to provide oral healthcare to the public under the social contract that legitimizes their professional autonomy [1, 2]. In return, they bear the obligation to improve the oral health of the communities they serve. As both healthcare professionals and members of society, dentists are expected to look beyond individual clinical care and actively address broader public oral health needs [3]. Despite the growing trend of viewing dentistry through a commercial and market-driven lens in an increasingly competitive environment [4], the ongoing global burden of oral diseases and persistent oral health inequities render the social responsibility of dentists more essential than ever [5].

As health professionals, dentists are perceived through various lenses—perceptions that evolve over time and vary depending on the observer [6]. For instance, Buldur & Armfield (2018) [7] investigated perceptions of dentists as professionals among dentists, dental students, and patients, revealing that all groups similarly recognized three core dimensions: status, human, and scientific factors. Interestingly, from children’s perspectives, dentists may be viewed as individuals who cause discomfort during treatment procedures, yet paradoxically, they are also perceived as protectors and pain relievers who provide care and comfort [8]. While perceptions of dentists vary depending on who is observing, when, and how they encounter the profession, one critical perspective remains constant: dentists serve as professionals who are integral members of society [3].

Although the necessity of social responsibility in dentistry is well recognized, the concept itself remains difficult to define in a single, unified manner. Social responsibility is a multifaceted construct, and the expected scope and depth of this responsibility may vary depending on cultural, institutional, and personal perspectives [9]. The social contract metaphor, often used to frame professional responsibility, has also been criticized for oversimplifying the complex relationship between professionals and society [10]. Nonetheless, considering that dentists are central to reducing oral health disparities, the importance of their social responsibility remains undeniable [11]. In light of these challenges, and drawing on literature on social responsibility in the health professions [12, 13], dental social responsibility is understood as the commitment and active engagement of dentists in promoting oral health not only at the individual level but also at the community, regional, and national levels—particularly among underserved and vulnerable populations.

Given this conceptual foundation, socially responsible dentists have consistently engaged in efforts to reduce oral health disparities. These efforts include providing preventive and therapeutic care to various underserved populations—such as those marginalized by age, race, ethnicity, or socioeconomic status—and actively participating in outreach and volunteer programs in rural and remote communities [14]. To nurture dentists who embody these socially responsible practices, dental education has continually identified social responsibility as a core competency for dental students [15, 16]. This focus seeks to prepare dental students not only to avoid being solely driven by commercial interests but also to advocate for and actively address the oral health needs of underserved populations. Such efforts help justify and sustain the social trust placed in dentists as privileged professionals.

However, despite the recognized importance and growing emphasis on social responsibility, comprehensive reviews examining the characteristics of such programs and their assessment methods remain scarce in the dental education literature. To address this gap, this study conducts a scoping review to identify and analyze educational programs explicitly designed to enhance dental students’ social responsibility. Specifically, this review aims to address the following research questions:

1. What are the key characteristics of educational programs designed to foster dental students’ social responsibility?
2. What methods have been employed to assess dental students’ social responsibility within these programs, and what outcomes have been reported?
3. What key components should be considered when designing educational programs to enhance dental students’ social responsibility?

The findings from this review are expected to inform the design, implementation, and evaluation of future educational programs, ultimately contributing to the cultivation of socially responsible dental professionals and the alleviation of oral health inequities at local and global levels.

## Methods

A scoping review methodology was employed to systematically map the scope and characteristics of existing literature on educational programs aimed at enhancing dental students’ social responsibility. Scoping reviews are particularly useful for identifying the breadth of available evidence and for understanding how research in a specific field has been conducted [17]. Accordingly, this approach was regarded as appropriate for mapping the landscape of such educational initiatives. The review is reported in accordance with the Preferred Reporting Items for Systematic Reviews and Meta-Analyses extension for Scoping Reviews (PRISMA-ScR) guidelines [18].

### Inclusion and exclusion criteria

Inclusion and exclusion criteria were structured according to the Population–Concept–Context (PCC) framework recommended for scoping reviews [19]. The participants (Population) of interest were undergraduate dental students. Students from other oral health professions (dental hygiene, dental therapy, dental nursing) and other health professions were excluded. The central concept investigated was educational programs explicitly aimed at promoting social responsibility. The context was limited to dental education, specifically within undergraduate dental curricula.

In addition to the PCC framework, studies were included if they met the following criteria: (1) original research articles published in peer-reviewed journals; (2) published in English; and (3) full-text availability. Studies were excluded if they were not original research (e.g., reviews, editorials, letters, notes, or book chapters), not published in English, or if the full text was not accessible. No date restrictions were applied to ensure comprehensive coverage of available literature. A detailed summary of these criteria is presented in Table 1.

**Table 1:** Inclusion and exclusion criteria

Category	Inclusion Criteria	Exclusion Criteria
Participants	Undergraduate dental students	Students from other oral health professions (e.g., dental hygiene, dental therapy, or dental nursing) Students from other health professions (e.g., medical, nursing, or allied health)
Concept	Educational programs explicitly aimed at enhancing social responsibility	Programs not focused on social responsibility or lacking clear relevance
Context	Dental education within predoctoral/undergraduate dental curricula	Postgraduate, continuing professional development, or non-dental educational contexts
Study type	Original research articles published in peer-reviewed journals	Reviews, editorials, letters, notes, book chapters
Language	English	Not English
Availability	Full text available	Full text not available

### Search strategy and study selection

A comprehensive literature search was conducted across three electronic databases: PubMed, Scopus, and Embase. These databases were selected for the following reasons: PubMed provided broad coverage of biomedical literature, including dental education; Scopus offered extensive multidisciplinary coverage across health sciences, social sciences, and education; and Embase captured additional European and international literature [20]. An initial set of search terms was developed by one of the authors, focusing on combinations of keywords related to “dental education” and “social responsibility.” The draft query was refined in consultation with a qualified health sciences librarian. The finalized search strategies for each database are detailed in Supplementary Table S1–S3 (see additional file 1). The search was performed on January 22, 2025.

Two reviewers independently conducted the study selection process. During both the title and abstract screening and the full-text review stages, the reviewers cross-checked each other’s decisions. Any discrepancies were resolved through discussion and consensus. Additionally, during the full-text review, both reviewers manually examined the reference lists of included studies to identify potentially relevant publications not captured in the initial database search. These references were retrieved, reviewed in full, and assessed for eligibility using the same inclusion and exclusion criteria.

### Data extraction

Key information was extracted using a predefined data extraction form developed for this review. Extracted data items included author, publication year, country, study aim, academic year of student participants, and characteristics of the educational program. Program characteristics were further classified according to the type of student activities (dental or non-dental), inclusion of reflective writing, and program duration. In addition, each study’s assessment methods and main outcomes related to social responsibility were systematically recorded. Two reviewers independently extracted data from all included studies. Their findings were then compared and integrated. When differences emerged, they were discussed until consensus was achieved. For instance, when disagreements arose in classifying student activities, procedures directly related to clinical dental care, such as oral examinations, fluoride applications, restorative treatments, and extractions, were categorized as dental activities by mutual agreement. A comprehensive data extraction table has been included in the Supplementary Material (see additional file 2). Based on the finalized dataset and informed by activity theory, a framework was developed to guide the design of educational strategies aimed at enhancing dental students’ social responsibility.

## Results

### Study selection

**Fig. 1:**
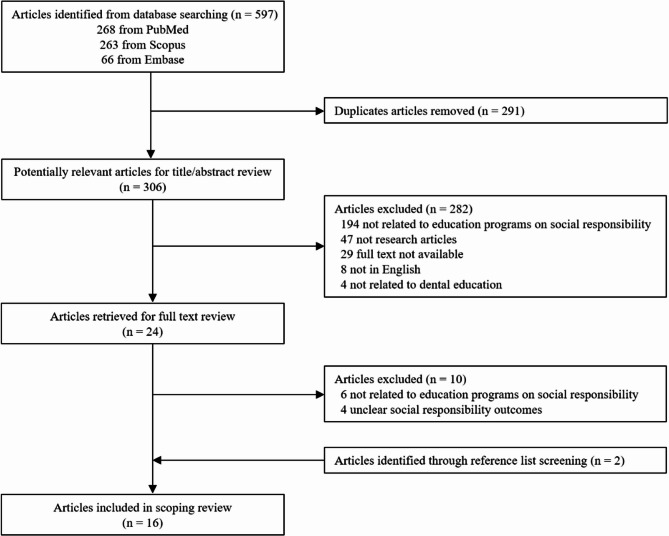
PRISMA flow diagram of study selection process

A total of 597 records were identified through database searches (PubMed = 268, Scopus = 263, Embase = 66). After removing 291 duplicates, 306 articles remained for title and abstract screening. Of these, 282 were excluded for the following reasons: unrelated to educational programs on social responsibility (*n* = 194), not original research articles (*n* = 47), not available in full text (*n* = 29), not published in English (*n* = 8), or not relevant to dental education (*n* = 4). This resulted in 24 articles being selected for full-text review. An additional 10 studies were excluded at this stage: not focusing on educational interventions related to social responsibility (*n* = 6), or lacking clearly defined social responsibility outcomes (*n* = 4). Two more articles were identified through reference list screening. In total, 16 studies met the inclusion criteria and were included in the final scoping review. Critical appraisal was not conducted, in line with the aim of mapping the breadth and scope of available evidence. A detailed overview of the study selection process is presented in Fig. 1.

### Study characteristics

The characteristics of the 16 included studies are summarized in Table 2, which presents the country of origin, study aim, participants’ academic year, and key components of the educational programs.

**Table 2:** Overview of educational programs fostering social responsibility in dental students

Author (Year)	Country	Aim	Academic Year (*n*)	Educational program		
				Student activities (Dental/Non-dental)	Reflective writing	Duration
Abllah et al. (2019) [21]	Malaysia	To assess how a community-learning program influenced final-year dental students	Final-year (64)	[Dental] • Oral health promotion using pamphlets, posters, videos, and games • Dental screening assistance • Educational setting: rural community	No	10-week preparation (3 hours/week) + 1-day implementation
Behar-Horenstein et al. (2015) [22]	USA	To explore the impact of service-learning on students’ beliefs about cultural competence, professionalism, disparities, and related values	Newly admitted (5)	[Non-dental] • Planning of preventive activities, leading of smoking cessation classes, community event assistance, and patients education in general health settings • Educational setting: homeless center, medical organization (underserved population), or county health department (migrant workers)	Yes	6 weeks
Bhayat et al. (2011) [23]	South Africa	To evaluate the impact of service-learning on the Phelophepa Health Care Train and Public Oral Health Facility on final-year dental students	Final-year (55)	[Dental] • Dental examinations, oral hygiene instructions, extractions, restorations, scaling • Educational setting: mobile healthcare train, public oral health facility	No	2 weeks (full-time)
Bhayat & Mahrous (2012) [24]	Saudi Arabia	To evaluate the impact of outreach activities on dental students	Third-year (32)	[Dental] • Dental examinations, oral hygiene and nutritional instructions • Educational setting: rehabilitation hospital, international primary school	No	4 hours
Brondani (2012) [25]	Canada	To thematically examine how the Professionalism and Community Service module fostered awareness of social responsibility	First- to fourth-year (190)	[Dental], [Non-dental] • Year 1: Conducting health promotion projects for vulnerable communities • Year 2: Engaging in health promotion and preclinical activities in long-term care facilities • Year 3: Providing preventive dentistry and preclinical care to children in inner-city schools • Year 4: Assisting in the delivery of clinical dental care to medically complex patients in hospitals and long-term care facilities	Yes	Integrated throughout a 4-year module
Brondani et al (2020) [26]	Canada	To design and evaluate a curriculum addressing substance use, queer health, and social responsibility	First-year (55)^a^	[Non-dental] • Participation in community-led workshops and presentations on substance use, queer health, and social responsibility, including pre-readings, videos, and interactive discussions	Yes	Three 2.5-hour sessions
Coe et al. (2015) [27]	USA	To evaluate the impact of a service-learning program on senior dental students’ attitudes toward community service	Final-year (105)	[Dental] Rotations through 12 external sites; specific activities not described	No	25 days on average
Dharamsi et al. (2010) [28]	Canada	To assess the impact of a new community service-learning option in a medical/dental curriculum	Not specified (36)	[Non-dental] • Health promotion projects targeting underserved groups (inner-city school children, frail elders in long-term care facilities, a women’s community centre, and a clinical preventive health care society) • Poster fair presentation	No	Not specified
Gadbury-Amyot et al. (2006) [29]	USA	To examine how a multifaceted ethics curriculum influences students’ attitudes toward service-learning, working in diverse communities, and disparities in care	First-year (103)^b^	[Dental] • Reading a book on cultural conflict • Oral screenings, oral hygiene instructions, fluoride varnish applications for children at a community center • Presentation of ethical dilemmas and collaborative solution-finding	Yes	7-week course
Holtzman & Seirawan. (2009) [30]	USA	To assess whether a community-based experience shapes students’ beliefs about the right of underserved populations to care and the responsibility of society and professionals to provide it	First-year (144)	[Dental] • Oral health promotion for elementary school children • Dental sealant placement, topical fluoride application in a mobile dental van	Yes	Across trimesters
Joury (2016) [31]	Syria	To describe the development and evaluation of an outreach dental public health programme	Third-year (400)	[Dental] • Comprehensive oral care for children in special needs centres (e.g., oral hygiene instruction, fluoride varnish application, sealant placement) • Research on dental public health • Development of health education materials and activities (e.g., puppet shows, interactive computer software, videos, songs)	No	Not specified
Mofidi et al. (2003) [32]	USA	To analyze students’ reflective essays to explore their community-based experiences, learning outcomes, and perceived benefits	Final-year (160)	[Dental] • Provision of dental care • Educational setting: public health facility (e.g., community health center, correctional institution, Native American health clinic), hospital or special needs facility	Yes	Two 4-week full-time rotations
Rubin (2004) [33]	USA	To evaluate a nondental community service program’s impact on students’ cultural competence and social responsibility	First-year (61)	[Non-dental] • Non-dental public health engagement (e.g., Pittsburgh AIDS task force, Hand-In-Hand Festival (for mentally and physically challenged children))	Yes	40 hours across 2 years
Rubin et al. (2008) [34]	USA	To assess changes in students’ attitudes, beliefs, and cultural competence after a two-year nondental community service program	First-year (126)	[Non-dental] • Non-dental public health engagement (e.g., volunteering with disaster relief groups, providing support to individuals with disabilities)	Yes	40 hours across 2 years
Suresan et al.(2019) [35]	India	To evaluate the effects of outreach programs on dental students’ academic and personal development, civic responsibilities, and gender-related differences	Final-year (100)	[Dental] • Oral health education, oral screenings, scaling, restorations, extractions • Educational setting: peripheral satellite centers, oral outreach treatment camps, public health installations • Visits to public health facilities (e.g., sewage treatment plant, water treatment plant)	No	Not specified
Witton & Paisi (2022) [36]	UK	To present key features of a social accountability program implemented for over ten years	Not specified (70–80/year)	[Dental] • Projects addressing oral health problems in underserved populations • Poster presentation and a year-end symposium	Yes	Not specified

^a^ Includes third-year dental hygiene students (*n* = 23)

^b^ Includes final-year dental hygiene students (*n* = 27)

Most studies were conducted in North America (United States and Canada; *n* = 10) [22, 25–30, 32–34], followed by Asia (*n* = 2) [21, 35], the Middle East (*n* = 2) [24, 31], Africa (*n* = 1) [23], and Europe (*n* = 1) [36]. This distribution illustrates that such educational efforts have been adopted across diverse global regions.

Participants were primarily undergraduate dental students, consistent with the inclusion criteria, and two programs additionally involved dental hygiene students through interprofessional or collaborative educational activities [26, 29]. Regarding academic levels, first-year [26, 29, 30, 33, 34] and final-year [21, 23, 27, 32, 35] dental students were most frequently involved (*n* = 5 each), followed by third-year students (*n* = 2) [24, 31]. Additionally, one longitudinal program spanned all four academic years [25], while another targeted newly admitted students [22]. Two studies did not specify the academic year of participants [28, 36].

The 16 included studies employed the following methodological approaches: quantitative (*n* = 6, 37.5%) [21, 23, 27, 30, 34, 35], qualitative (*n* = 5, 31.3%) [22, 25, 26, 32, 33], and mixed-methods (*n* = 5, 31.3%) [24, 28, 29, 31, 36].

### Student activities and program duration

All included educational interventions shared a community-based pedagogical approach. Dental students engaged directly with communities and collaborated with local stakeholders [21–25, 27–36]. They also occasionally involved community members as educators within the educational sessions [26]. This underscored that the community served as a central context and partner in the educational process.

Dental-related activities were predominant [21, 23–25, 27, 29–32, 35, 36], encompassing both preventive and clinical care. Specifically, programs included oral health promotion activities utilizing various materials (e.g., pamphlets, posters, videos, and interactive activities) [21, 23–25, 29–31, 35, 36], as well as dental screenings [21, 23, 24, 29, 35]. Additionally, some programs involved fluoride varnish and sealant application [25, 29–31], as well as restorative and surgical procedures such as extractions [23, 25, 32, 35]. These interventions targeted vulnerable groups such as inner-city school children, older adults in long-term care facilities, hospital patients with complex needs, and individuals in special-needs institutions [21, 23–25, 28, 30–32, 35, 36]. Non-dental activities, while not involving direct clinical dental procedures, addressed broader social determinants of health [22, 25, 26, 28, 33, 34]. These activities included health promotion projects targeting underserved groups, such as disaster relief volunteering and disability support services [34]. Several programs combined dental and non-dental activities to comprehensively address the health needs of targeted communities [25]. Reflective writing was integrated into over half of the reviewed programs (*n* = 9) [22, 25, 26, 29, 30, 32–34, 36]. It served as a structured method to facilitate students’ critical analysis and deeper comprehension of their professional roles and responsibilities within society.

The duration of educational programs varied substantially across the included studies, ranging from short-term interventions to multi-year curricular integrations. The shortest interventions included single-day or brief sessions, such as a one-time 4-hour activity [24] or a series of three 2.5-hour sessions [26]. Several programs adopted intermediate durations, conducted over weeks [21–23, 27, 29, 32], ranging from two to ten weeks. A few studies extended activities over longer academic timelines, incorporating community service totaling approximately 40 hours distributed across two academic years [33, 34] or implementation across academic trimesters [30]. Notably, one comprehensive program was longitudinally integrated throughout all four academic years [25]. This program involved weekly half-day sessions in the first year, followed by term-specific engagements in subsequent years. However, four of the included studies did not specify the precise duration of the program [28, 31, 35, 36].

### Assessment methods and outcomes

Table 3 summarizes the assessment methods and key outcomes reported in the included studies. To evaluate the development of dental students’ social responsibility, most studies employed either surveys [21, 23, 24, 27–30, 34–36] or reflective writing [22, 25, 26, 29, 31–33]. Surveys—used in both pre/post [27, 29, 34, 35] and post-only [21, 23, 24] designs—commonly assessed changes in students’ awareness, attitudes, and intentions. Representative findings included increased awareness of community needs [21, 23, 24, 36], stronger sense of responsibility to serve the community [21, 23, 24, 27, 34], and greater intent to incorporate community service into future professional plans [21, 23, 24, 28, 29, 34]. Several studies reported statistically significant improvements on validated scales measuring constructs such as connectedness [27] and civic responsibility [35]. However, Holtzman and Seirawan (2009) [30] reported a decline in students’ sense of responsibility for underserved populations over the course of their first year.

**Table 3:** Summary of assessment methods and main outcomes related to social responsibility

Author (Year)	Assessment methods	Main outcomes (quantitative and qualitative)
Abllah et al. (2019) [21]	Survey (post-intervention)	[Quantitative] Representative survey items: • Increased awareness of community needs (98.3% agree) • Increased sense of responsibility to serve the community (100% agree) • Increased awareness of role in community (100% agree) • Intention to integrate community service into future plans (87.9% agree)
Behar-Horenstein et al. (2015) [22]	Reflective writing Interview	[Qualitative] Representative student comment: “The experience also inspired me to work towards treating more patients with Medicaid and those who are in underserved communities.”
Bhayat et al. (2011) [23]	Survey (post-intervention)	[Quantitative] Representative survey items: • Increased awareness of community needs (96% agree) • Increased sense of responsibility to serve the community (85% agree) • Increased awareness of role in community (94% agree) • Intention to integrate community service into future plans (74% agree)
Bhayat & Mahrous (2012) [24]	Survey (post-intervention)	[Quantitative] Representative survey items: • Increased awareness of community needs (100% agree) • Increased sense of responsibility to serve the community (90% agree) • Increased awareness of role in community (86% agree) • Intention to integrate community service into future plans (90% agree) [Qualitative] Representative response to open-ended question: “I think we should go back and provide more services to these types of communities.”
Brondani (2012) [25]	Reflective writing	[Qualitative] Representative thematic finding: Students considered “their contribution to the classroom and the community, their views on diversity and sensitive issues, and their responsibilities as future health care providers.” These aspects collectively constitute the core element of social responsibility.
Brondani et al. (2020) [26]	Reflective writing	[Qualitative] Representative student comment: “This session gave me the opportunity to hear from that side of the community and what they want from a socially responsible dentist—the other side is the dental professional recognizing [their] privilege and giving back what they think society needs.”
Coe et al. (2015) [27]	Survey (retrospective pre-intervention and post-intervention)	[Quantitative] Statistically significant increase in connectedness construct: • It is my responsibility to take some real measures to help others in need. • It is important to me to gain an increased sense of responsibility from participating in community service. • I feel an obligation to contribute to the community.
Dharamsi et al. (2010) [28]	Survey Focus group interview Interview	[Quantitative] Survey results: “Students mostly expected they would provide care in the future to underserved population groups.” [Qualitative] Contents under the theme “Witnessing”: Community service-learning was perceived to raise awareness that a vulnerable community “desperately needs good physicians and good dentists.”
Gadbury-Amyot et al. (2006) [29]	Survey (pre-intervention and post-intervention) Reflective writing	[Quantitative] Survey results: Statistically significant increase in attitudes toward volunteerism over time, indicating greater willingness to volunteer. [Qualitative] Representative student comments: “Yes, make money but also be aware that we have an obligation to ALL individuals in society.”
Holtzman & Seirawan (2009) [30]	Survey (3 rounds)	[Quantitative] Survey results: Statistically significant decline in students’ attitudes regarding dentist and student responsibility for caring for the underserved over the course of their first year.
Joury (2016) [31]	Reflective writing	[Qualitative] Representative student comments: • “Syrian Smiles provided us with an academic framework to articulate our willingness to serve our community; I want to be engaged in more community serving activities.” • “I will open my heart and clinic to children with special needs; I don’t have any fear or concern to provide them with the care they need.”
Mofidi et al. (2003) [32]	Reflective writing	[Qualitative] Contents under the theme “Commitment to service”: • A desire to specialize in pediatric dentistry “to reach the lower socioeconomic pediatric population” • Providing voluntary treatment to residents of nursing homes • “incorporating care for those less fortunate in my dental practice”
Rubin (2004) [33]	Reflective writing	[Qualitative] Representative student comments: • “I have always been interested in politics and this experience made me think more about my involvement in the community when I graduate from school.” • “I knew all of the facts but in the back of my mind I was still a little uncomfortable with the thought of volunteering at this event [AIDS walk]… It was a great chance for me to meet and chat with the people from the community… I have already participated in the AIDS walk again.”
Rubin et al. (2008) [34]	Survey (retrospective pre-test and post-test)	[Quantitative] Statistically significant increase in scores on the community-related scale: • I picture myself volunteering my dental services to members of the community. • Dentists should reserve a percentage of their office time to treat low-income families. • Disregarding legal mandates, dentists have an obligation to do community service.
Suresan et al. (2019) [35]	Survey (pre-intervention and post-intervention)	[Quantitative] Statistically significant increase in scores on the civic responsibility domain.
Witton & Paisi (2022) [36]	Not explicitly specified; based on student feedback data	[Quantitative] Survey results: • 88% reported increased awareness of population health issues and social inequalities at a community level. • 87% reported that they now feel confident to work with and engage the community outside the clinical environment. [Qualitative] Representative student comment: “It’s really made me think more about inclusion, and has encouraged me to put more effort into thinking about what I can do as a dental practitioner in the future to make dental access for these more vulnerable groups easier.”

In addition to quantitative assessments, qualitative approaches such as reflective writing were also widely employed to capture students’ internalization of social responsibility [22, 25, 26, 29, 31–33]. Analysis of these reflections revealed common themes such as commitment to serve vulnerable populations [31–33], increased awareness of social inequities [26, 36], and deeper understanding of the dentist’s societal role [25, 26, 28]. Students often expressed intentions to pursue socially oriented careers [32], or integrate community care into their future practices [36].

### Key components of educational programs

**Fig. 2:**
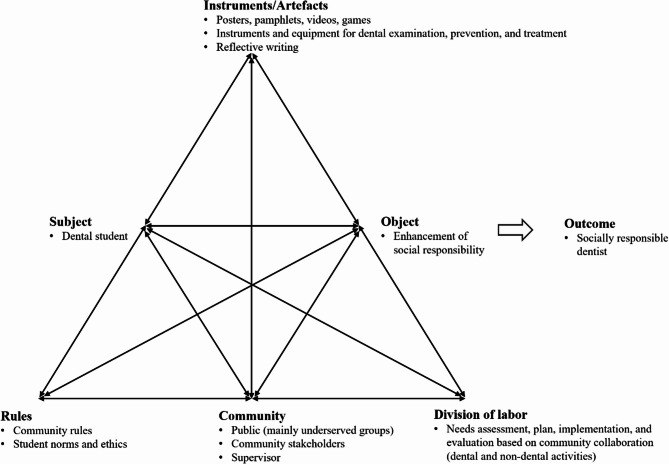
Conceptual framework for community-based education to enhance dental students’ social responsibility, based on Engeström’s activity system. The subject is the dental student engaged in activities oriented toward the object—enhancement of social responsibility. The activity is mediated by instruments/artefacts (e.g., educational materials, reflective writing, clinical equipment), governed by contextual rules (e.g., community rules, student ethics), and embedded within a community (e.g., underserved populations, local stakeholders, supervisors). The division of labor reflects collaborative processes of needs assessment, planning, implementation, and evaluation. The intended outcome is the development of a socially responsible dental professional. This framework supports experiential and reflective learning grounded in authentic community engagement

Based on the analysis of the 16 studies and drawing on activity theory, a framework is proposed to guide the implementation of community-based education aimed at enhancing social responsibility. Activity theory, originating from Vygotskian sociocultural theory and expanded by Engeström, can provide a framework for understanding how students learn through structured participation in activities [37]. The activity system comprises several interrelated elements—subject, object, instruments/artefacts, rules, community, and division of labor—that collectively produce meaningful outcomes [37]. Figure 2 presents the proposed framework based on Engeström’s activity system, which highlights key components of community-based educational programs that effectively promote dental students’ social responsibility. Within this framework, the subject represents dental students engaged in community-oriented activities, aiming toward an object of enhanced social responsibility, leading to an outcome of socially responsible dental practitioners. Instruments/artefacts include educational resources (e.g., posters, pamphlets, videos, games) and clinical instruments and equipment. The rules encompass community-specific norms and guidelines addressing professional and ethical student behavior. The community primarily comprises underserved populations, community stakeholders, and supervising faculty, collaboratively involved in co-constructing educational experiences. The division of labor clarifies roles and responsibilities related to collaborative needs assessment, program planning, implementation, and evaluation. Collectively, these elements support experiential learning closely linked to reflection and community engagement [38].

## Discussion

This scoping review identified 16 studies that implemented educational programs aimed at enhancing dental students’ social responsibility. Community-based education emerged as the predominant pedagogical approach, often accompanied by reflective writing. Surveys and reflective writing consistently revealed improvements in students’ awareness, attitudes, and intentions related to social responsibility. These findings underscore the importance of embedding structured opportunities within dental curricula to cultivate social responsibility as a core professional value.

A notable finding of this review is the central role of community-based education, also referred to as service-learning [27], in fostering social responsibility. In these programs, students delivered meaningful services within communities, while the communities provided authentic learning opportunities. This reciprocal model benefits both learners and community members and allows students to move beyond traditional environments, such as classrooms or dental hospitals, into real-world settings [39]. A particularly impactful aspect of this reciprocal model is when community members themselves become educators, sharing their lived experiences directly with students [26]. For example, listening to personal stories from individuals with experiences of addiction, queer individuals, and people living with HIV provides students with opportunities to identify community needs more directly and engage in critical thinking, rather than remaining passive recipients of knowledge. As highlighted in a recent review of community-based dental education [40], such programs enhance clinical competencies, academic knowledge, and interpersonal skills, while also fostering social responsibility—an outcome aligned with the present review.

While the enhancement of clinical skills and knowledge remains an important outcome, the educational significance of community-based education lies in the participatory process. Students are encouraged to identify community needs, engage with key stakeholders, and collaboratively plan, implement, and evaluate interventions [41]. These initiatives need not be confined to clinical contexts. Several programs in this review effectively engaged preclinical students [26, 29, 30, 33, 34], including those newly admitted [22], and incorporated non-dental activities addressing broader social determinants of health [22, 25, 26, 28, 33, 34]. Importantly, many of these programs were directed toward underserved populations within local communities, reinforcing students’ understanding of health disparities and their responsibility to address them. Considering these points, dental schools should avoid limiting such initiatives based on academic level or activity type. Instead, they should provide students with early and sustained opportunities to engage directly with communities, identify real-world challenges, and contribute to their resolution. These efforts are essential for cultivating socially responsible dental professionals who are equipped to respond to the oral health needs of diverse and underserved populations. Furthermore, in an evolving healthcare environment that increasingly emphasizes integrated care [42], such participatory and collaborative experiences can lay the foundation for dental professionals to enact social responsibility as active members of the broader health care team.

Both surveys and reflective writing demonstrated meaningful improvements in dental students’ social responsibility. Survey instruments were employed in 10 of the 16 studies. Although surveys enable the measurement of constructs related to social responsibility and allow for the identification of changes in perception, several important considerations emerged from this review. When interpreting the results, social desirability bias should be considered, as students may respond in ways they perceive to be socially acceptable rather than reflecting their true attitudes or intentions [43]. In addition, the lack of standardized instruments for measuring social responsibility within the context of dental education limits the comparability of findings across programs. Despite these methodological limitations, most studies reported improvements in students’ social responsibility following educational interventions. The development of validated, context-specific instruments for assessing social responsibility in dental education would enhance the comparability of future studies and support the design of more evidence-based educational programs.

Particularly, reflective writing serves as both a learning and evaluative tool within community-based educational programs. Through post-experience reflections, students articulated their intentions to serve and provide care for marginalized groups, including “patients with Medicaid,” [22] “underserved communities,” [22] “children with special needs,” [31] “lower socioeconomic pediatric population,” [32] and “those less fortunate.” [32]. These reflections offer insight into students’ evolving values and their deepening awareness of health disparities, as emphasized by Eyler (2002) [44]. In this context, reflective writing not only enables students to critically analyze their experiences but also fosters a more profound understanding of their professional responsibilities to society as future dentists. From a pedagogical perspective, this approach in community-based education facilitates transformative learning, where students’ existing beliefs, perspectives, and mindsets are challenged through experiences in different contexts, leading them to think critically and reinterpret their understanding, ultimately resulting in shifts in their professional identity and worldview [45]. It also provides faculty with meaningful opportunities to assess students’ professional development and to deliver formative feedback that enhances the overall learning experience. Accordingly, incorporating structured reflection should be considered essential when implementing community-based education in dental curricula [46].

Based on the analysis of the 16 studies and activity theory, the framework presented in Fig. 2 can be utilized when designing educational programs to enhance social responsibility. This framework suggests that effective community-based programs should ensure: (1) clear role definitions and collaborative partnerships between students, faculty, and community stakeholders; (2) appropriate educational and clinical resources tailored to community needs; (3) explicit guidelines that balance professional standards with community-specific contexts; and (4) structured reflection processes that enable students to critically examine their experiences and professional development. By addressing these interconnected elements, dental educators can create more systematic and impactful learning experiences that cultivate socially responsible practitioners.

While the findings of this scoping review provide valuable insight into existing educational strategies for promoting social responsibility, there are two critical areas that require further investigation. First, there is a need for longitudinal evaluation of educational outcomes throughout the dental curriculum. Only one study included in this review adopted a three-point survey design to track changes in students’ attitudes over time [30]. Notably, this study found a decline in students’ sense of responsibility toward underserved populations during their first year. This counterintuitive finding underscores the importance of examining how social responsibility evolves during dental training, and whether specific curricular experiences strengthen or erode this commitment. Future studies should explore these dynamics across diverse educational contexts and seek to identify underlying factors contributing to such trends. Second, there is a need to examine whether the development of social responsibility during dental school leads to sustained professional behaviors after graduation. As Brondani (2012) [25] noted, participating in community-based education does not guarantee that all students will truly adopt or sustain a sense of social responsibility. This finding highlights the need to investigate whether such educational experiences lead to lasting behavioral change after graduation. Similarly, the conceptual framework presented in Fig. 2 should be interpreted with the understanding that the outcome of ‘socially responsible dentist’ is not an assured result of educational interventions, as individual results will vary based on student engagement and contextual factors.

### Limitations

This review has several limitations. First, while most included studies reported improvements in students’ sense of social responsibility, this trend may reflect a publication bias toward positive outcomes. Programs with less favorable or null findings may be underrepresented in the literature. Additionally, there are students whose perceptions of social responsibility do not change despite program participation, and further research is needed to understand what characteristics and experiences influence these outcomes and what alternative educational programs might be required for them. Second, although community-based education emerged as a key strategy, the outcomes were predominantly student-focused and primarily derived from survey-based data. To gain a more comprehensive understanding of program effectiveness, future research should incorporate the perspectives of faculty, community stakeholders, and patients. Finally, the inclusion of English-language publications only may have limited the scope of this review, excluding potentially diverse and locally adapted educational programs reported in other languages.

## Conclusion

This scoping review identified and analyzed key characteristics of educational programs designed to enhance dental students’ social responsibility. Community-based education emerged as central to these programs. Findings underscore the importance of providing dental students with structured opportunities to engage directly with communities, particularly underserved populations, and to reflect critically on these experiences. Although further research is needed to examine the long-term impacts of such educational interventions, dental curricula should continue to develop, implement, and evaluate community-based programs that cultivate social responsibility. This approach reinforces the role of dental students as privileged future professionals who are accountable to society.

## Supplementary Information

Supplementary Material 1.

Supplementary Material 2.

## Data Availability

No datasets were generated or analysed during the current study.

## Abbreviations

PRISMA-ScR: Preferred reporting items for systematic reviews and meta-analyses extension for scoping reviews
PCC: Population–concept–context
