# Supplementary material for: Educational strategies to enhance dental students’ social responsibility: a scoping review
Source: BMC Med Educ. 2025 Oct 2;25:1335. doi: 10.1186/s12909-025-07941-x (PMC12492727; doi:10.1186/s12909-025-07941-x)
Supplement: Supplementary file 2 — Supplementary Material 2. [file 12909_2025_7941_MOESM2_ESM.docx]

**Table S1. PubMed search strategy**

| **Search number** | **Query** | **Results** |
| --- | --- | --- |
| 1 | ((dental education[MeSH Terms]) OR (dental student[MeSH Terms])) OR (dental[Text Word] AND (educat*[Text Word] OR student*[Text Word] OR learn*[Text Word] OR teach*[Text Word])) | 68,244 |
| 2 | (social responsibility[MeSH Terms]) OR (social responsibilit*[Text Word]) | 28,081 |
| 3 | (social accountability[MeSH Terms]) OR (social accountabilit*[Text Word]) | 26,220 |
| 4 | (social obligation[MeSH Terms]) OR (social obligatio*[Text Word]) | 26,086 |
| 5 | #1 AND (#2 OR #3 OR #4) | 268 |

**Table S2. Embase search strategy**

| **Search number** | **Query** | **Results** |
| --- | --- | --- |
| 1 | 'dental education'/exp OR 'dental education' OR 'dental student'/exp OR 'dental student' OR ((dental NEAR/3 (educat* OR student* OR learn* OR teach*)):ti,ab,kw) | 44,544 |
| 2 | 'social responsibility'/exp OR 'social responsibilit*':ti,ab,kw OR 'social obligatio*':ti,ab,kw OR 'social accountabilit*':ti,ab,kw | 6,863 |
| 3 | #1 AND #2 | 66 |

**Table S3. Scopus search strategy**

| **Search number** | **Query** | **Results** |
| --- | --- | --- |
| 1 | ( dental AND ( educat* OR student* OR learn* OR teach* ) ) AND ( "social responsibilit*" OR "social obligatio*" OR "social accountabilit*" ) | 263 |
